# Comparison of bioimpedance and oesophageal Doppler cardiac output monitoring during abdominal aortic surgery

**DOI:** 10.1186/cc10825

**Published:** 2012-03-20

**Authors:** HK Jørgensen, J Bisgaard, T Gilsaa

**Affiliations:** 1 Littlebaelt Hospital Kolding, Denmark

## Introduction

Abdominal aortic surgery is a high-risk procedure. Cardiac output monitoring allowing haemodynamic optimisation may reduce the complication rate. Minimally invasive, continuous techniques are preferable. Cardiac output using oesophageal Doppler has been validated in several studies, showing good agreement with the gold standard. The aim of this study was to assess the degree of correlation and agreement between cardiac output measured by oesophageal Doppler and bioimpedance obtained from an endotracheal tube.

## Methods

Twelve patients scheduled for elective abdominal aortic surgery were included. Patients were intubated with an ECOM™ endotracheal tube (ConMed Corporation, NY, USA) which was connected to the ECOM™ monitoring system. An oesophageal Doppler probe (CardioQ™; Deltex Medical, UK) was inserted, connected to the CardioQ-ODM™ monitoring system and correct positioning verified. Simultaneous determination of cardiac output by ODM and ECOM™ was performed before and after cross-clamping of the aorta.

## Results

**Figure 1:**
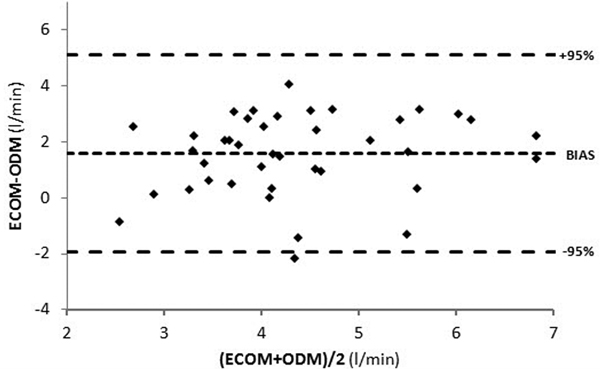


Cardiac output ranged from 1.4 to 13.1 l/minute. Linear regression is represented by the equation *y *= 0.*30x *+ 2.2 and the correlation coefficient *r*^2 ^= 0.15. The bias was +1.5 l/minute with 95% limits of agreement between -2.1 and 5.1 l/minute (Figure 1).

## Conclusion

Using the CardioQ™ as a reference, the ECOM™ system cannot be recommended as a clinical cardiac output measurement technique in abdominal aortic surgery, due to its poor correlation and wide limits of agreement.

